# Evolutionary and dynamic constraints converge on a conserved coupling sector in thermally adapted GH1 β-glucosidases

**DOI:** 10.1371/journal.pcbi.1014879

**Published:** 2026-10-27

**Authors:** Li-Ting Zheng, Yi Li, Xian He, Lian-Tao Yang, De-Rui Zhao, Meng-Ting Liu, Peng Sang, Li-Quan Yang

**Affiliations:** 1 College of Pharmacy, Dali University, Dali, China; 2 College of Agriculture and Biological Science, Dali University, Dali, China; 3 Key Laboratory of Bioinformatics and Computational Biology, Department of Education of Yunnan Province, Dali University, Dali, China; 4 Co-Innovation Center for Cangshan Mountain and Erhai Lake Integrated Protection and Green Development of Yunnan Province, Dali University, Dali, China; 5 College of Mathematics and Computer Science, Dali University, Dali, China; Shiraz University, IRAN, ISLAMIC REPUBLIC OF

## Abstract

How protein sequences evolve to maintain functional conformational dynamics under thermal stress remains an important question in molecular evolution. Thermophilic enzymes are often considered to achieve stability primarily through enhanced structural rigidity, yet thermal adaptation may also involve the preservation or reorganization of correlated motions across the protein scaffold. Here, we compared three glycoside hydrolase family 1 β-glucosidases—BsBgl1A, 9UPT, and LQBG8—that share a conserved (β/α)_8_ TIM-barrel fold but display distinct optimal temperatures of approximately 45, 65, and 70 °C, respectively. By integrating comparative structural analysis, three independent 1 μs molecular dynamics simulations at 300, 325, and 350 K, neural relational inference of residue-level dynamic coupling, and family-level coevolutionary analysis, we identified distinct dynamic strategies associated with their thermal phenotypes. All three enzymes retained their global compactness and principal secondary-structure framework across the simulated temperatures, indicating that differences in RMSD and residue flexibility did not reflect large-scale unfolding. BsBgl1A showed the strongest temperature sensitivity, with increased structural deviation and residue mobility at 350 K. Its inferred interaction network exhibited a non-monotonic response, characterized by pronounced overcoupling at 325 K followed by reduced connectivity at 350 K. In contrast, 9UPT maintained relatively restrained residue dynamics and a highly reproducible coupling topology across temperatures. LQBG8 combined substantial local conformational flexibility with persistent long-range coupling and retained the highest directed network density at 350 K. Across the three enzymes, the βα5–βα7 region repeatedly emerged as a shared structural and dynamic center. Coevolutionary couplings were also enriched in TIM-barrel-associated regions that spatially converged on this sector. These findings indicate that thermal adaptation in GH1 β-glucosidases is not adequately explained by uniform structural rigidification. Instead, enzymes with the same conserved fold may adopt distinct organizations of correlated motion, while greater thermal adaptation is associated with the persistence or reorganization of a βα5–βα7-centered dynamic coupling pattern under elevated temperature. The correspondence between dynamic coupling and coevolutionary enrichment is consistent with long-term evolutionary constraint on this structural sector, although it does not establish direct selection on the network as an independent functional unit. Our results therefore provide a testable framework in which thermostability reflects a balance among local stabilization, regional flexibility, and the resilience of higher-order dynamic organization.

## Author summary

GH1 β-glucosidases are widely distributed enzymes that perform similar catalytic functions across organisms adapted to very different temperatures. Although their overall structures are highly conserved, how thermal adaptation is encoded in their internal dynamics remains unclear. In this study, we combine long-timescale molecular dynamics simulations, Neural Relational Inference, and evolutionary sequence analysis to compare three GH1 β-glucosidases with distinct temperature optima. We find that all three enzymes preserve the canonical TIM-barrel fold, but respond to heating through different patterns of flexibility and residue–residue communication. BsBgl1A shows the strongest temperature-dependent disruption of its dynamic network, whereas 9UPT maintains a restrained and reproducible response. LQBG8 remains locally flexible while preserving extensive long-range coupling at high temperature. Notably, residues located mainly within the fifth to seventh βα modules repeatedly emerge as a shared region of dynamic and evolutionary constraint. These results suggest that thermal adaptation is not achieved through uniform rigidification. Instead, heat-adapted GH1 enzymes may preserve function by balancing local flexibility with the maintenance or reorganization of coordinated motions in structurally important regions.

## 1. Introduction

How protein sequences evolve to preserve functional integrity under extreme environmental conditions remains a central question in molecular evolution [1–3]. Proteins operating at high temperatures face persistent thermodynamic challenges, including enhanced thermal fluctuations, weakened noncovalent interactions, and increased conformational entropy [1,2,4]. Despite these destabilizing forces, many thermophilic enzymes maintain remarkable catalytic efficiency and structural integrity under conditions that rapidly inactivate their mesophilic counterparts [2]. Understanding how evolutionary processes reconcile thermal destabilization with sustained function is fundamental to our understanding of molecular adaptation [1,3].

Classical models of thermophilic adaptation have largely emphasized static structural features, such as increased hydrophobic packing, additional salt bridges, or reduced loop flexibility, as primary determinants of thermostability [4,5]. While these mechanisms undoubtedly contribute to protein robustness, they provide an incomplete explanation [2,6]. Proteins are intrinsically dynamic systems, and enzymatic function depends not only on maintaining a folded structure but also on sustaining coordinated internal motions across multiple spatial and temporal scales [6,7]. Excessive rigidification can stabilize a fold yet impair catalytic efficiency, indicating that functional adaptation requires a balance between stability and flexibility [8–10]. Whether evolution preserves this balance by constraining dynamic organization, rather than solely reinforcing local structural elements, remains poorly understood [3,9].

Recent advances in molecular simulations and network-based analyses have highlighted the importance of long-range residue interactions and internal correlated interaction pathways in shaping protein dynamics [11–14]. Correlated residue motions and interaction-network representations have been used to describe how perturbations may be distributed across protein structures [11, 12]. However, it remains unclear whether such dynamic interaction networks are merely emergent physical properties of folded structures or whether they represent evolutionarily conserved organizational units [13–15]. Although coevolutionary analyses have identified residue pairs under long-term evolutionary constraint [3,15–17], and molecular dynamics simulations have elucidated temperature-dependent conformational behavior [18], these perspectives have largely remained disconnected. How evolutionary pressures act on dynamic interaction networks, rather than on isolated residues or static contacts, is still an open question [3,14,15].

Here, we address this gap by comparing three GH1 β-glucosidases with highly conserved folds but distinct biochemical optima. BsBgl1A and LQBG8 were identified from hot-spring metagenomic resources, whereas 9UPT was included as an intermediate-temperature representative with an experimentally resolved structure. All three enzymes belong to glycoside hydrolase family 1 and adopt the canonical (β/α)_8_ TIM-barrel fold [19], yet they display strikingly different thermal optima and stability profiles [2,18]. Specifically, BsBgl1A, a GH1 family β-glucosidase isolated from Hehua Hot Spring in Tengchong, Yunnan, China, was characterized by our experimental data to maintain good stability under alkaline conditions (pH 8.5) with an optimal catalytic temperature of 45 °C, demonstrating application potential in moderately warm and alkaline environments [20]; The enzyme represented by PDB entry 9UPT exhibits an intermediate thermal phenotype, with an optimal temperature of approximately 65 °C and an optimal pH of 5.5. LQBG8, another GH1 β-glucosidase identified from a metagenomic library from the same geothermal region as BsBgl1A, displays greater thermal adaptability, with an optimal reaction temperature of approximately 70 °C and an optimal pH of 5.6, while retaining high catalytic activity under elevated-temperature and mildly acidic conditions [21]. The combination of a conserved overall fold and divergent biochemical properties provides an informative framework for examining whether differences in thermal adaptation are associated with distinct organizations of internal protein dynamics rather than with major changes in global topology [22,23]. We therefore integrate temperature-resolved molecular dynamics simulations with neural relational inference of residue-level interaction networks to characterize how statistically coupled motions are reorganized under increasing thermal stress [24,25]. These dynamic patterns are further examined in the context of family-level coevolutionary constraints to assess whether residues participating in persistent long-range coupling also exhibit evidence of long-term evolutionary association. By relating temperature-dependent dynamic organization to structural and evolutionary conservation, this integrative framework enables us to test the hypothesis that enhanced thermal adaptation in GH1 β-glucosidases is associated with the persistence or reorganization of specific global coupling architectures, rather than with uniform rigidification of the protein structure [26].

Rather than presenting dynamic interaction networks as a new concept, our objective is to examine a more focused evolutionary hypothesis: whether family-level evolutionary constraints are associated not only with individual residues and pairwise contacts, but also with recurrent higher-order patterns of correlated motion. By integrating molecular dynamics simulations, neural relational inference, and family-level coevolutionary analysis, we assess whether temperature-dependent dynamic coupling patterns spatially converge with evolutionarily constrained regions within the conserved GH1 TIM-barrel scaffold. This integrative analysis does not establish that network architecture is an independent target of natural selection. Instead, it provides a framework for evaluating whether the maintenance or reorganization of higher-order dynamic coupling may represent an additional consequence of evolutionary constraint alongside residue conservation, coevolving residue pairs, structural packing, and allosteric organization.

## 2. Materials and methods

### 2.1. Structural model construction and system preparation

The amino acid sequences of BsBgl1A and LQBG8 were obtained from previously reported thermophilic β-glucosidases isolated from hot-spring metagenomic resources. Initial structural models were reconstructed using the SWISS-MODEL homology-modeling server based on experimentally resolved GH1 β-glucosidase templates [27].

For BsBgl1A, the model was generated using the crystal structure of a GH1 β-glucosidase from Exiguobacterium antarcticum B7 as the template (PDB ID: 5DT5.1.A), with a sequence identity of 77.75%, GMQE of 0.92, and QMEANDisCo global score of 0.88 ± 0.05. For LQBG8, the model was generated using a GH1 β-glucosidase A structure from Halothermothrix orenii as the template (PDB ID: 4PTV.1.A), with a sequence identity of 46.97%, GMQE of 0.82, and QMEANDisCo [28] global score of 0.80 ± 0.05.

For comparison, the experimentally resolved crystal structure of 9UPT, a GH1 β-glucosidase from Acetivibrio thermocellus, was obtained directly from the Protein Data Bank (PDB ID: 9UPT, chain A) [29]. Because 9UPT represents an experimentally determined structure, no additional homology modeling was performed. The deposited structure was inspected for structural completeness and used as the initial conformation for subsequent molecular dynamics simulations after standard structural preparation.

The final structures comprised 444 residues for BsBgl1A, 447 residues for 9UPT, and 441 residues for LQBG8 and were used as the starting conformations for subsequent molecular dynamics simulations.

To assess the structural consequences of replacing the original AlphaFold2 (AF2)-derived models with homology models, the corresponding AF2 and homology-model structures of BsBgl1A and LQBG8 were directly compared. Residue correspondence for RMSD calculations was restricted to positions with identical residue numbers and residue identities in both structures, yielding 444 matched residues for BsBgl1A and 441 matched residues for LQBG8. Global backbone RMSD was calculated after least-squares superposition of backbone atoms (N, Cα, and C). Global fold similarity was independently evaluated using TM-align as implemented in the tmtools package, and TM-scores normalized by the lengths of both the AF2 and homology models were reported.

For region-specific comparison, the conserved TIM-barrel core comprising β1–β8 and α1–α8 was first superimposed using backbone atoms. Consistent with the subsequent structural and dynamic analyses, the eight β–α structural regions (βα1–βα8) were defined according to the corresponding β-strand-to-α-helix segments. Regional backbone RMSDs were then calculated after TIM-barrel core alignment without additional region-specific refitting, and residue-wise Cα displacements were evaluated to identify localized structural differences. Detailed region definitions and structural comparison results are provided in S1 Table and S1 Fig.

To evaluate structural conservation between the three enzymes, structure-based alignment was performed using the Dali server. The resulting alignments were visualized with ESPript 3.0 [30,31], enabling comparative analysis of sequence similarity in the context of secondary-structure correspondence. These models served as starting conformations for subsequent molecular dynamics simulations.

### 2.2. Molecular dynamics simulations

All-atom molecular dynamics (MD) simulations were performed using GROMACS 2021.5 with the CHARMM36 force field, which provides improved accuracy for protein backbone conformations and side-chain interactions and has been extensively validated for long-timescale simulations of globular proteins [32,33].

Each protein was solvated in a dodecahedral box containing TIP3P water molecules with a minimum solute-wall distance of 1.0 nm [34]. Counterions were added to neutralize the systems, followed by the addition of NaCl to a final concentration of 150 mM. The protonation states of ionizable residues were assigned using the H++ server according to the experimentally reported optimal pH of each enzyme. BsBgl1A, 9UPT, and LQBG8 were protonated at pH 8.5, 5.5, and 5.6, respectively, to approximate their corresponding biochemical environments.

Energy minimization was first carried out using the steepest descent algorithm, followed by 100 ps NVT equilibration and 400 ps NPT equilibration at the target temperature [35]. Independent simulations were performed separately at 300 K, 325 K, and 350 K.

For each temperature, three independent replicas of 1 μs were performed with different initial velocity distributions. The temperature was maintained using the velocity-rescaling thermostat, and pressure was controlled at 1 bar using the Parrinello–Rahman barostat [36]. Covalent bonds involving hydrogen atoms were constrained using the LINCS algorithm with a 2fs integration timestep [37]. Long-range electrostatic interactions were calculated using the particle mesh Ewald method [38]. Coordinates were saved every 10 ps for subsequent analyses.

### 2.3. Trajectory preprocessing and conformational analyses

All molecular dynamics trajectories were first preprocessed to remove periodic boundary artifacts and rigid-body motions by aligning each frame to the initial structure based on backbone Cα atoms. Conformational stability and global structural deviations were quantified by calculating the root-mean-square deviation (RMSD) of backbone atoms (N, Cα, and C) using MDTraj [39]. Protein compactness was assessed by calculating the radius of gyration (Rg) throughout each trajectory. RMSD and Rg trajectories were further divided into five equal-duration blocks to evaluate time-dependent stability and convergence across the simulations.

Local conformational flexibility was assessed by computing per-residue Cα root-mean-square fluctuations (RMSF), providing a residue-level measure of temperature-dependent mobility. Secondary structure evolution along the trajectories was analyzed using the DSSP algorithm as implemented in GROMACS, enabling quantification of temperature-induced changes in secondary structure composition and persistence. All conformational metrics were calculated relative to the initial predicted structure to ensure consistency across temperatures and simulation replicas.

To further capture temperature-dependent reorganization of residue–residue interactions, dynamic contact maps were constructed based on Cα–Cα distances using the ConAn package. Residue pairs were defined as being in contact when their Cα atoms were within a cutoff distance of 10 Å. Contact persistence and survival probabilities were subsequently derived from time-resolved contact maps, providing a dynamic representation of interaction stability across temperature conditions. These analyses collectively provide a quantitative foundation for subsequent network inference and evolutionary interpretation.

### 2.4. Network inference from molecular dynamics trajectories

To characterize long-range dynamic interactions within thermophilic β-glucosidases, we employed neural relational inference (NRI), a graph-based deep learning framework that infers latent interaction structures directly from trajectory data. Originally developed to uncover underlying physical interaction graphs in dynamical particle systems, NRI has recently been adapted to biomolecular simulations due to its ability to capture both direct and indirect couplings that are difficult to resolve using conventional correlation- or contact-based approaches.

In the NRI framework, residues are represented as nodes in a graph, while their dynamic relationships are encoded as probabilistic edges. Rather than relying on static spatial proximity, NRI integrates both spatial and kinetic information, enabling the inference of interaction patterns that best explain the observed temporal evolution of molecular trajectories. Specifically, the model consists of an encoder–decoder architecture: the encoder infers a latent interaction graph from trajectory segments, and the decoder predicts future system dynamics conditioned on this inferred graph. Before feature extraction, periodic boundary artifacts were removed from all molecular dynamics trajectories using GROMACS. Each trajectory frame was subsequently fitted to the corresponding reference structure using backbone atoms to minimize global translation and rotation. Protein conformations were extracted from each 1 μs trajectory at intervals of 200 ps, yielding approximately 5,000 frames per trajectory. The aligned all-atom trajectories were then projected onto Cα representations for NRI-based network inference. Positional and velocity features were calculated from the aligned Cα trajectories so that the model inputs primarily represented relative residue motions within the protein rather than variations caused by arbitrary coordinate-frame selection. To retain residue-level dynamic information while maintaining computational tractability, each residue was treated as an individual graph node. The resulting networks contained 444 nodes for BsBgl1A, 447 nodes for 9UPT, and 441 nodes for LQBG8. Each node was described by six normalized features comprising its three-dimensional position and velocity coordinates. This full residue-resolution representation avoided biases associated with the arbitrary selection of coarse-grained nodes and enabled analysis of statistically coupled motions throughout the entire protein structure.

Model training followed the protocol described by Zhu et al. and Zhao et al [24,40]. The encoder–decoder architecture was trained using the Adam optimizer with an initial learning rate of 0.0005. The learning rate was reduced by a factor of 0.5 every 200 epochs, and training was conducted for a total of 500 epochs using a batch size of one. The model with the lowest validation loss was retained for subsequent inference. The model used four latent edge types, with the first class representing the absence of an interaction. A sparsity prior of 0.91, 0.03, 0.03, and 0.03 was applied to the four edge classes, respectively. The positional and velocity features together constituted six input dimensions for each residue. For each possible directed residue pair, the trained encoder produced a probability distribution over the four latent edge classes. Because the first class represented the absence of an interaction, the total interaction probability from residue *i* to residue *j* was defined as


$$\begin{array}{c} p_{ij}=\text{1}-p_{ij}(noedge). \end{array}$$

The interaction probability of each directed residue pair was averaged across all trajectory sequences in the corresponding dataset. The resulting probabilities were arranged into an *N × N* directed interaction matrix, with self-connections excluded. These probabilities quantify statistical dependencies between residue motions and should not be interpreted as direct physical contacts, causal communication pathways, or energy-transfer routes.

To reduce the contribution of weak or noisy interactions to network visualization, directed edges with mean interaction probabilities below 0.1 were excluded from the thresholded weighted networks. Edge-probability distributions were evaluated using histograms and cumulative distribution functions constructed from the mean probabilities of all possible directed residue pairs. Sensitivity to threshold selection was assessed over probability thresholds ranging from 0.02 to 0.30. At a given threshold, the retained-edge fraction was calculated as the proportion of all possible directed residue pairs, excluding self-connections, whose mean interaction probabilities were equal to or greater than the selected threshold.

Network organization was quantitatively characterized using directed network density, total node degree, and weighted node strength. Directed network density was calculated as


$$\begin{array}{c} D=\frac{E}{N(N-\text{1}\text{0})}, \end{array}$$

where *E* is the number of retained directed edges and *N* is the number of residue nodes. The total degree of a node was defined as the sum of its incoming and outgoing edges. Weighted node strength was defined as the sum of the probabilities of all retained incoming and outgoing edges associated with that node. Average total degree and average weighted node strength were calculated by averaging the corresponding node-level values across all residues.

To assess the consistency of the inferred networks, the trained encoder was applied to the held-out test dataset with model parameters fixed. Agreement between the training and held-out networks was evaluated using Pearson and Spearman correlations of directed edge probabilities, the Jaccard index for the top 1% highest-probability edges, Spearman correlation of residue-level total weighted strength, and Spearman correlation of βα-module-level interaction strength.

Residue-level interaction matrices were further aggregated according to the eight sequential βα structural modules, βα1–βα8, of the GH1 TIM-barrel fold. Module-level incoming and outgoing interaction strengths were calculated by summing the corresponding residue-level edge probabilities and normalizing by the number of residues within each module. The resulting βα-module-level interaction matrices and directed network diagrams were visualized using Cytoscape through the Py4Cytoscape interface. The βα modules were arranged in a fixed sequential order across all network diagrams. Node size reflected total module connectivity, whereas edge transparency represented inferred interaction strength.

### 2.5. Family-level coevolutionary analysis

To characterize evolutionary constraints acting on β-glucosidase sequences at the family level, we assembled a structurally and functionally representative dataset of glycoside hydrolase family 1 (GH-1) enzymes. Experimentally resolved β-glucosidase structures were retrieved in April 2025 from the InterPro database (entry IPR001360), with corresponding Protein Data Bank identifiers listed in S2 Table. An initial set of 323 GH-1 structures was collected.

To ensure comparability with the two thermophilic β-glucosidases investigated in this study, structures were filtered based on sequence length (400–500 amino acids) and global structural similarity. Specifically, only structures satisfying both a backbone root-mean-square deviation (RMSD) of less than 3 Å and a TM-score greater than 0.80 relative to either BsBgl1A or LQBG8 were retained, resulting in a final dataset of 178 representative GH1 β-glucosidase structures for subsequent multiple sequence alignment and coevolutionary analysis. Pairwise sequence identities within this dataset were calculated using Biopython [41], while structural similarities were evaluated using ProDy [42], ensuring broad evolutionary coverage while maintaining fold-level consistency.

Family-level coevolutionary constraints were inferred using the transformer-based protein language model esm-msa-1b, which captures residue–residue statistical dependencies from multiple sequence alignments without explicit reliance on structural information. Multiple sequence alignments corresponding to the selected GH-1 enzymes were provided as input, and coevolutionary coupling scores were computed using the contact_prediction workflow implemented in the ESM framework. The resulting coupling matrices quantify evolutionary constraints between residue positions across the β-glucosidase family, reflecting long-term selective pressures acting on sequence organization.

These coevolutionary coupling patterns were subsequently used as an evolutionary reference to assess whether residues participating in dynamic interaction networks identified from molecular dynamics simulations are subject to conserved evolutionary constraints. Importantly, coevolutionary analyses were performed independently of any dynamic or structural information derived from simulations, enabling an unbiased comparison between evolutionary coupling and dynamically inferred interaction architectures.

## 3. Results

### 3.1. Evolutionary constraints define a conserved core in GH1 β-glucosidases

To establish the evolutionary and structural context underlying high-temperature adaptation, we compared the three-dimensional architectures of the thermophilic β-glucosidases BsBgl1A and LQBG8 with that of the structurally related GH1 β-glucosidase represented by PDB entry 9UPT. Despite their different sequence backgrounds and biochemical properties, all three proteins adopt the canonical glycoside hydrolase family 1 (GH1) fold, consisting of an eightfold repeating β–α arrangement that forms a typical (β/α)₈ TIM-barrel architecture (Fig 1A–1C). In this scaffold, eight parallel β-strands constitute the central barrel, whereas the surrounding α-helices form an outer structural shell. The corresponding β-strands and α-helices were designated β1–β8 and α1–α8, respectively.

**Fig 1:**
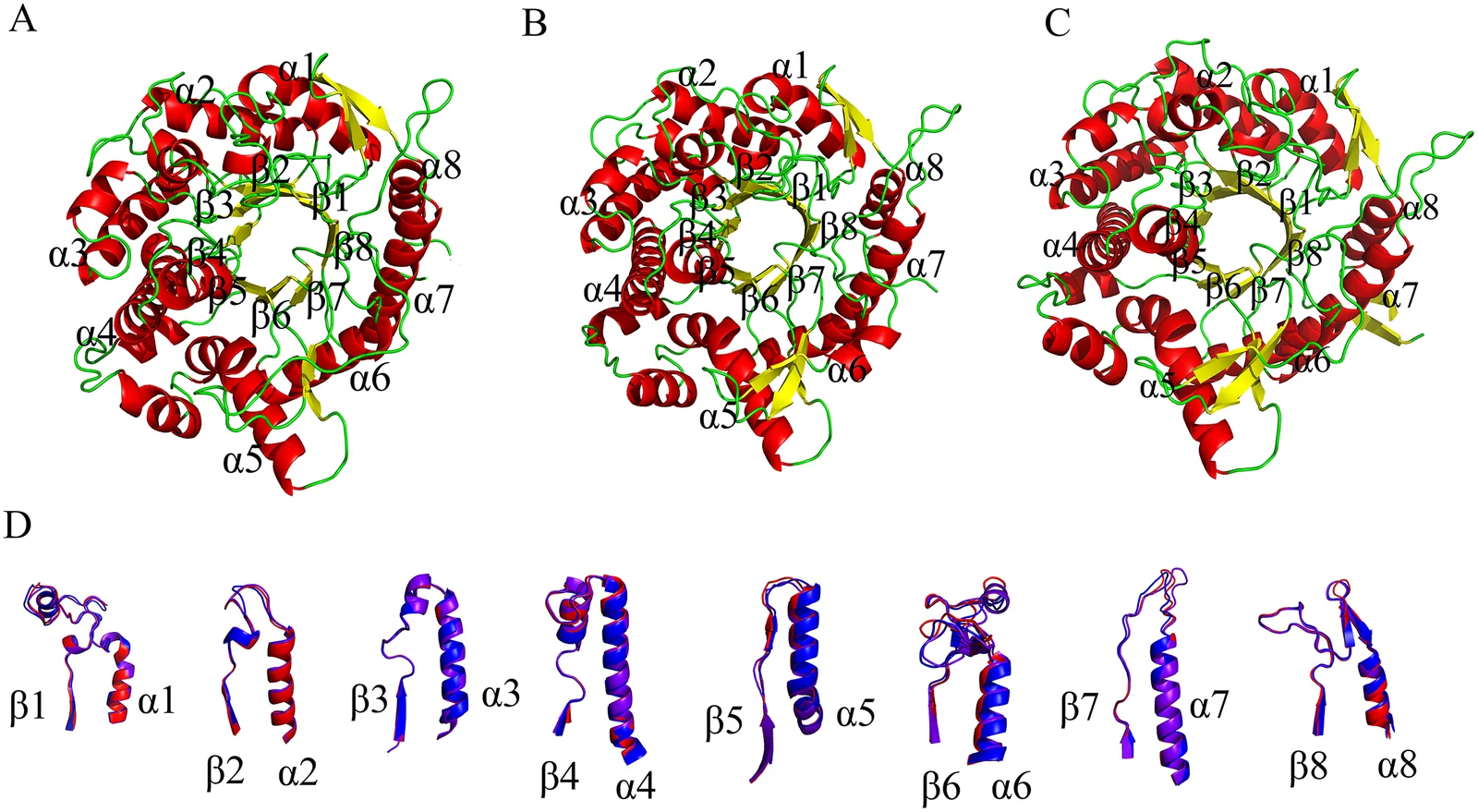
Conserved (β/α)₈ TIM-barrel architecture and localized structural divergence among three GH1 β-glucosidases. **(A)** Cartoon representation of BsBgl1A, showing the canonical GH1 (β/α)₈ TIM-barrel fold. The eight parallel β-strands forming the central barrel and the surrounding α-helices are labeled β1–β8 and α1–α8, respectively. **(B)** Cartoon representation of the GH1 β-glucosidase represented by PDB entry 9UPT, displayed in the same orientation as BsBgl1A to facilitate direct structural comparison. **(C)** Cartoon representation of LQBG8, showing a highly similar overall TIM-barrel organization. **(D)** Separate superposition of the eight corresponding β–α structural units from BsBgl1A, 9UPT, and LQBG8. BsBgl1A, 9UPT, and LQBG8 are shown in blue, purple, and red, respectively. The close overlap of the β-strands and adjacent α-helices demonstrates strong conservation of the barrel-forming framework, whereas structural deviations are mainly localized to solvent-exposed loops and terminal regions of the secondary-structure elements.

Comparison of the overall structures revealed a high degree of conservation in the spatial organization of the TIM-barrel scaffold. In particular, the relative arrangement of the eight β–α structural units was maintained across BsBgl1A, 9UPT, and LQBG8, indicating that the global fold is under strong evolutionary constraint. Consistent with this observation, structure-based superposition of BsBgl1A and LQBG8 yielded a backbone RMSD of only 0.693 Å, despite their moderate sequence identity of 40.59%. The marked contrast between sequence divergence and structural similarity suggests that substantial amino acid substitutions can be accommodated without disrupting the overall GH1 fold.

To further resolve the distribution of structural conservation, the eight individual β–α units from BsBgl1A, 9UPT, and LQBG8 were extracted and superimposed separately (Fig 1D). The β-strands and the major portions of the adjacent α-helices showed close spatial overlap among the three enzymes, particularly within the central barrel-forming regions. By contrast, the largest deviations were observed at the peripheral connecting segments, including the loops preceding the β-strands and the terminal regions of several α-helices. These differences were especially apparent in structural units containing extended or irregular loop conformations, such as the β1–α1, β5–α5, β6–α6, β7–α7, and β8–α8 regions.

These observations indicate that structural diversification within GH1 β-glucosidases occurs predominantly in solvent-exposed peripheral regions, whereas the β-strand framework and the core β–α organization of the TIM barrel remain highly conserved (Fig 2). The central barrel therefore appears to constitute an evolutionarily constrained structural framework required for maintaining catalytic geometry and global fold integrity, while peripheral loops provide structurally permissive regions in which sequence insertions, deletions, and conformational remodeling can occur.

**Fig 2:**
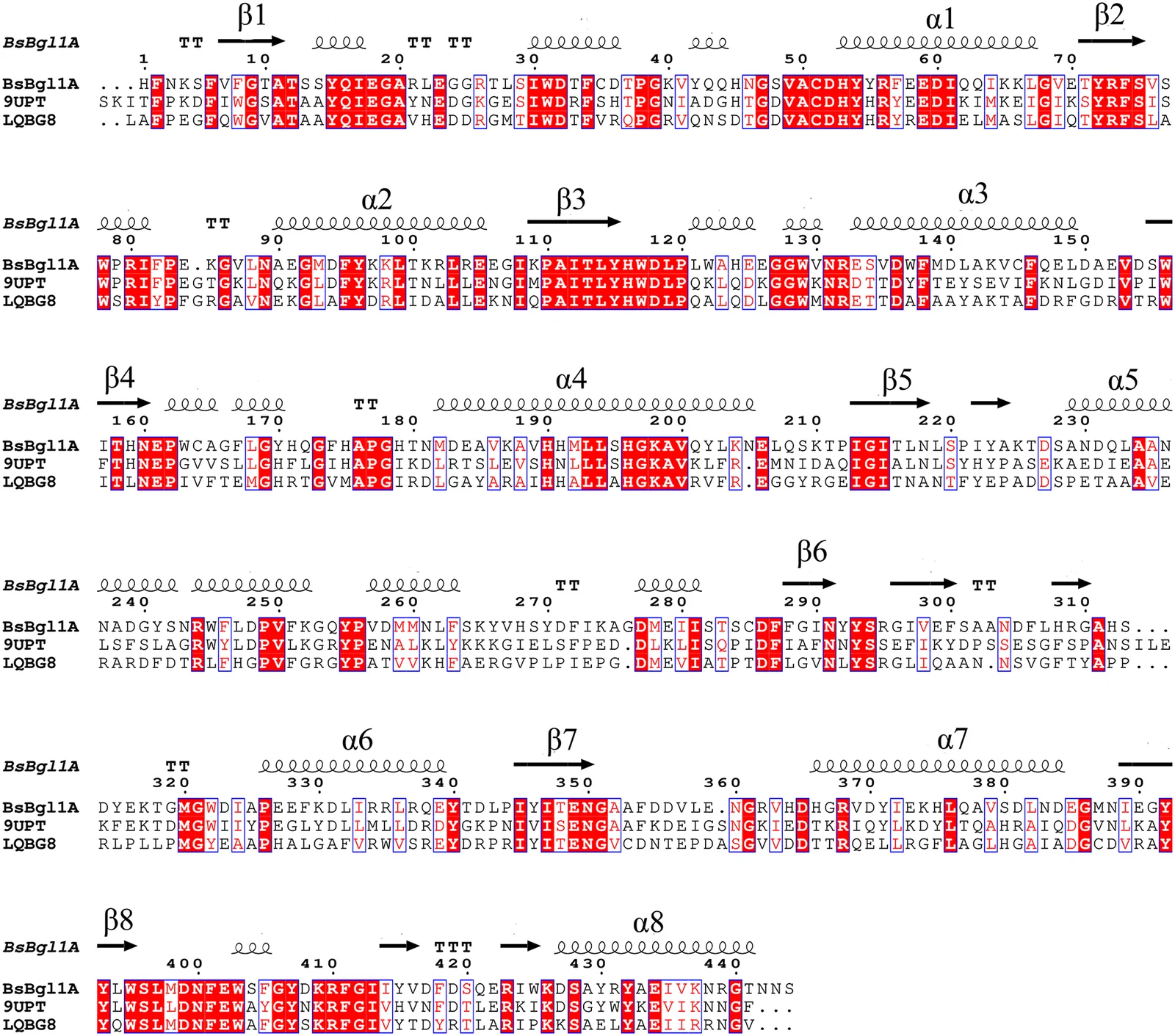
Structure-guided sequence alignment of BsBgl1A, 9UPT, and LQBG8. The alignment was generated based on structural superposition and annotated with secondary structure elements derived from the respective models. Identical residues are shown in white text on a red background, and conserved substitutions are shown in red text on a white background. β-strands and α-helices are indicated by arrows and cylinders, respectively, and are numbered sequentially according to their positions in the TIM-barrel scaffold. Regions corresponding to βα and αβ connecting loops are indicated, highlighting pronounced sequence variability in βα loops relative to the conserved αβ segments.

### 3.2. High-temperature adaptation correlates with selective stabilization of the TIM-barrel core

Having established the highly conserved TIM-barrel architecture of BsBgl1A, 9UPT, and LQBG8, we next examined whether this structural conservation was accompanied by similar dynamic responses to thermal perturbation. Temperature-resolved molecular dynamics simulations were therefore performed at 300, 325, and 350 K, with three independent 1 μs trajectories generated for each enzyme at each temperature. Global conformational stability was first evaluated using backbone root-mean-square deviation (RMSD) and radius of gyration (Rg) analyses (S2 and S3 Figs).

The RMSD trajectories revealed distinct dynamic behaviors among the three enzymes. At 300 and 325 K, BsBgl1A and 9UPT generally sampled lower backbone deviations than LQBG8, whereas LQBG8 rapidly reached a higher RMSD plateau, indicating a greater departure from its initial structure. At 350 K, the RMSD values of all three proteins increased, but the magnitude and temporal development of this response differed. BsBgl1A displayed a pronounced progressive increase in backbone deviation, indicating enhanced temperature sensitivity. LQBG8 maintained the highest or near-highest RMSD values over much of the trajectory, whereas 9UPT showed the smallest overall deviations and the most restrained response to elevated temperature. Block-wise analysis confirmed these trends, with increasing RMSD across successive trajectory segments being particularly evident for BsBgl1A at 350 K.

Despite these differences in backbone displacement, the Rg values of all three enzymes remained within a relatively narrow range throughout the simulations. Only modest temperature- and time-dependent variations were observed, and no persistent large-scale expansion was detected. Thus, the elevated RMSD values reflect conformational rearrangement within the folded ensemble rather than global unfolding or collapse of the TIM-barrel scaffold. In particular, the combination of relatively high RMSD and stable Rg in LQBG8 suggests that this enzyme accesses alternative compact conformations while preserving its overall fold.

Residue-level Cα RMSF analysis further resolved the spatial distribution of thermal fluctuations (Fig 3). The three enzymes shared several flexible regions, most notably the N-terminal segment and a series of solvent-exposed loop regions. However, the magnitude and temperature dependence of these fluctuations differed substantially. BsBgl1A exhibited relatively low overall flexibility at 300 K, followed by a moderate increase at 325 K and a pronounced amplification at 350 K. The strongest temperature-induced peaks were concentrated around the N-terminal region and several peripheral segments in the middle and C-terminal portions of the sequence, indicating that thermal perturbation is absorbed primarily by discrete flexible elements rather than being uniformly distributed throughout the structure.

**Fig 3:**
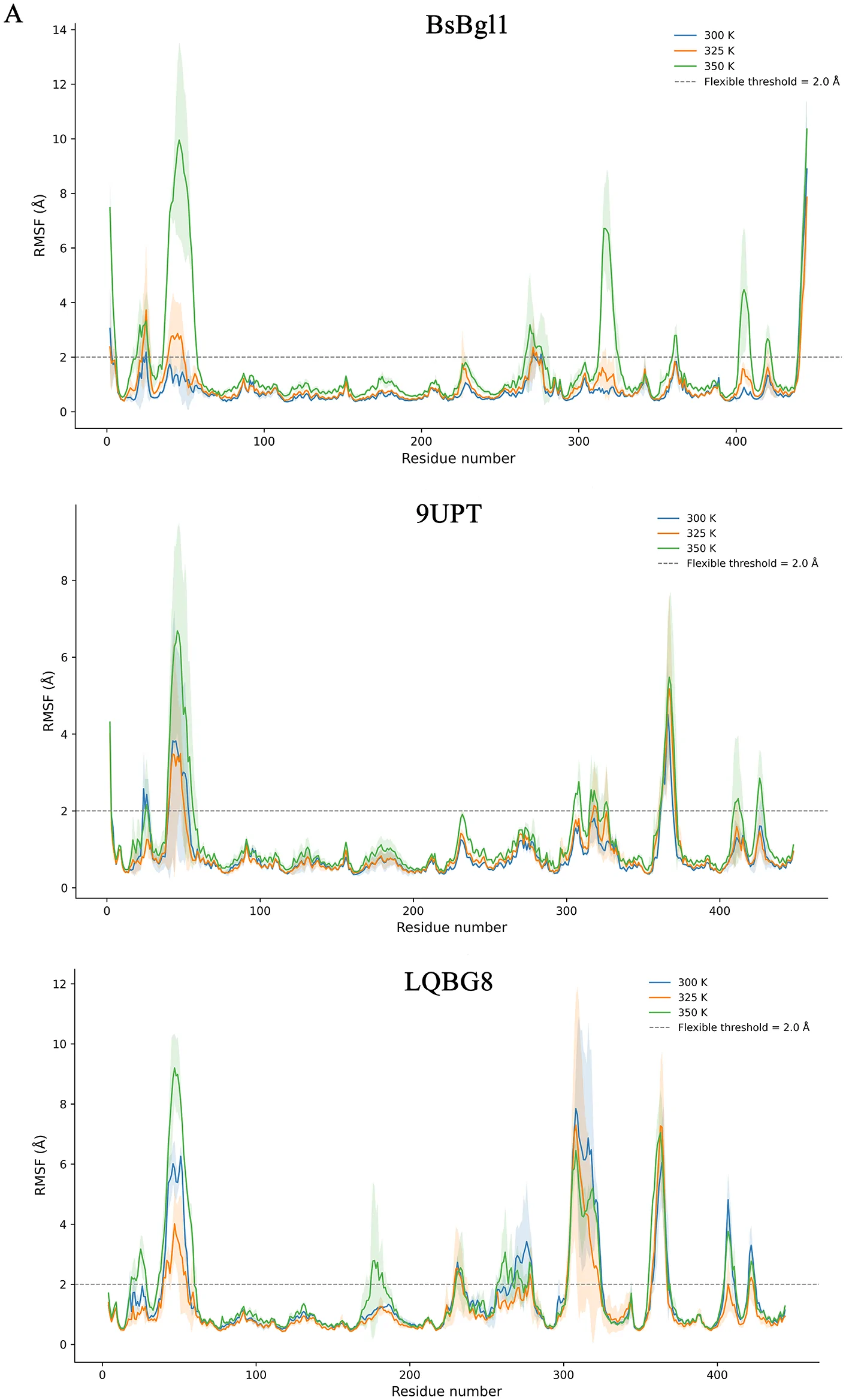
Temperature-dependent residue-level flexibility of BsBgl1A, 9UPT, and LQBG8. Residue-wise Cα root-mean-square fluctuation (RMSF) profiles calculated from three independent 1 μs molecular dynamics simulations at 300 K, 325 K, and 350 K. The upper, middle, and lower panels show BsBgl1A, 9UPT, and LQBG8, respectively. The mean RMSF profiles at 300, 325, and 350 K are shown in blue, orange, and green, respectively, and the corresponding shaded regions represent the standard deviation among independent simulations. The horizontal dashed line denotes an RMSF threshold of 2.0 Å and was used to identify highly flexible residues. Elevated fluctuations are predominantly localized to the N-terminal and solvent-exposed loop regions, whereas most barrel-associated secondary-structure elements remain comparatively restrained.

Among the three enzymes, 9UPT showed the lowest residue-level fluctuations at 350 K and the smallest proportion of residues exceeding the 2.0 Å flexibility threshold. Most of its secondary-structure-associated regions remained below this threshold, with elevated fluctuations restricted to a limited number of loops and connecting segments. These observations identify 9UPT as the most dynamically restrained of the three structures under the simulated conditions.

LQBG8 displayed a different dynamic regime. Its mean RMSF was already higher at 300 K, and a comparatively large fraction of residues exceeded 2.0 Å, indicating substantial intrinsic flexibility under near-ambient conditions. Although the mean RMSF decreased slightly at 325 K, it increased again at 350 K, accompanied by pronounced peaks in the N-terminal region and several loop-rich segments around the middle and C-terminal portions of the protein. Therefore, LQBG8 should not be considered uniformly rigid. Instead, it appears to combine a preserved compact fold with extensive localized conformational mobility. Quantitative comparison of the mean RMSF and the percentage of highly flexible residues supported these conclusions (S4 Fig): 9UPT consistently exhibited the most restricted thermal response, BsBgl1A showed the clearest temperature-dependent increase, and LQBG8 maintained the greatest basal and high-temperature flexibility.

To determine whether these fluctuations were accompanied by disruption of regular secondary structure, we next analyzed the time-dependent secondary-structure composition and residue-wise secondary-structure occupancy (Fig 4). Across all three enzymes, the principal α-helical and β-strand elements remained broadly preserved between 300 and 350 K, consistent with the stable Rg profiles and indicating that none of the proteins underwent global unfolding. The β-strands forming the central TIM-barrel scaffold were particularly persistent, supporting the idea that the barrel core represents a strongly conserved and dynamically stable structural unit.

**Fig 4:**
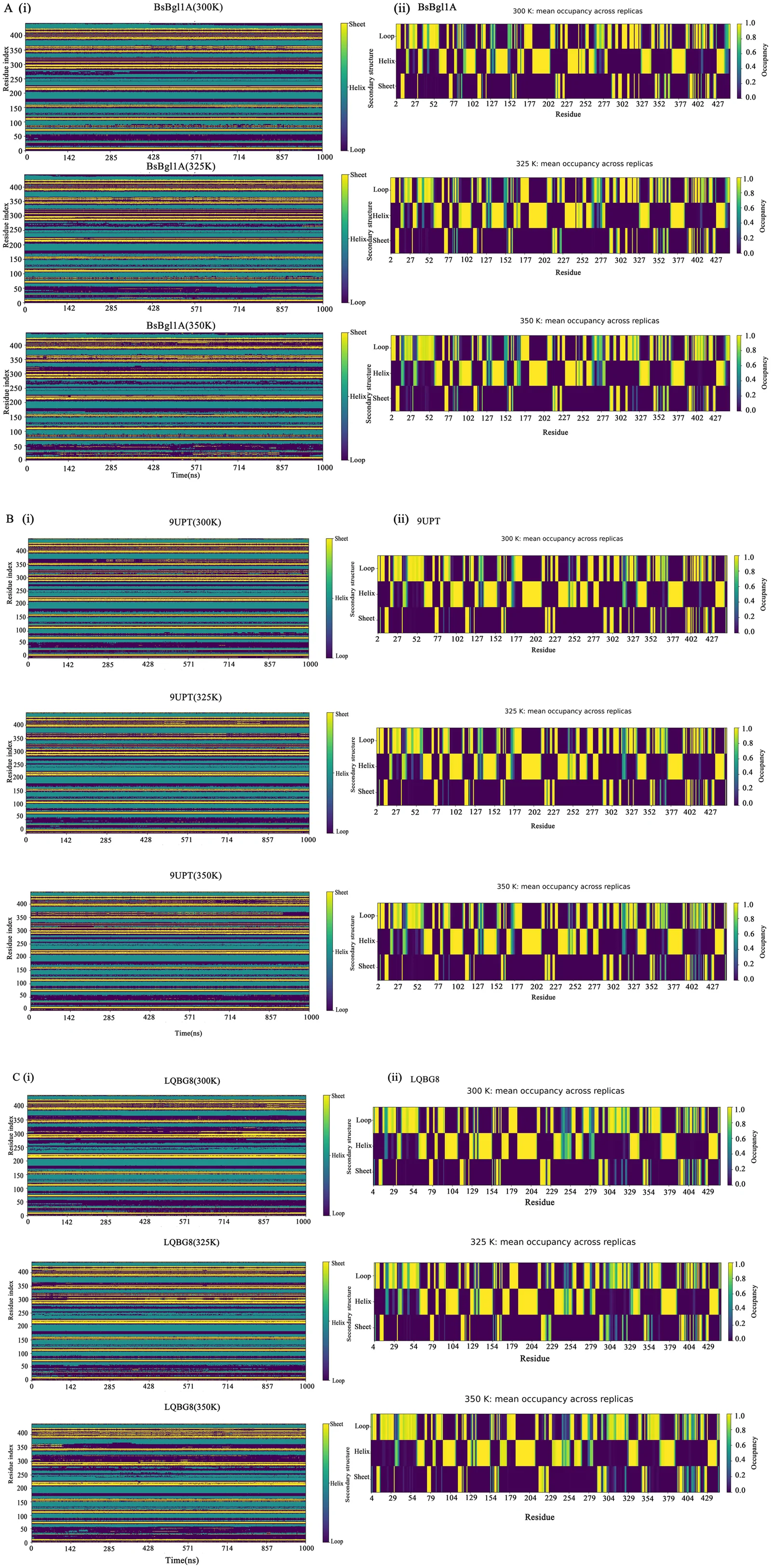
Temperature-dependent secondary-structure dynamics of BsBgl1A, 9UPT, and LQBG8. (A–C) Secondary-structure analyses of BsBgl1A, 9UPT, and LQBG8, respectively, at 300, 325, and 350 K. (i) Time-resolved secondary-structure assignments for individual residues during the 1 μs portion of the molecular dynamics trajectories. The horizontal axis represents simulation time and the vertical axis represents residue index. Colors indicate loop, α-helical, and β-strand conformations. (ii) Mean residue-wise occupancy of loop, helix, and strand states calculated across the three independent simulations at each temperature. The color scale represents the fractional occupancy of each secondary-structure state. The persistence of the principal α-helical and β-strand elements indicates preservation of the overall TIM-barrel architecture, whereas local temperature-dependent transitions are primarily confined to peripheral and loop-rich regions.

Nevertheless, local differences in secondary-structure persistence were evident. BsBgl1A showed increased switching between helical, strand, and loop conformations in several peripheral regions at elevated temperature, in agreement with its strong RMSF increase at 350 K. LQBG8 also exhibited appreciable local secondary-structure heterogeneity, especially in regions corresponding to major RMSF peaks, but its central barrel-forming elements remained largely retained. By contrast, 9UPT displayed the most continuous and reproducible secondary-structure patterns across temperatures, with comparatively limited redistribution among secondary-structure states.

To determine whether these temperature-dependent structural adjustments were accompanied by changes in active-site organization, we quantified the distance between the two catalytic glutamate residues throughout the simulations (S6 Fig). Across all three enzymes and all simulated temperatures, the mean catalytic-residue distance remained largely stable, with only minor temperature-dependent shifts and substantial overlap among the three independent replicas. These results indicate that the observed differences in conformational dynamics were not accompanied by pronounced disruption of the catalytic-site geometry. Thus, the subsequent differences in NRI-derived dynamic coupling patterns occurred within conformational ensembles that retained the overall spatial organization of the catalytic residues.

Taken together, these results demonstrate that thermal adaptation in GH1 β-glucosidases cannot be described simply in terms of uniform global rigidity. All three enzymes preserve the compact TIM-barrel fold, but they differ in how thermal motion is distributed within that framework. 9UPT exhibits the strongest overall dynamic restraint, BsBgl1A undergoes a marked temperature-dependent increase in localized flexibility, and LQBG8 maintains substantial conformational plasticity while avoiding large-scale structural expansion. Thus, preservation of the GH1 scaffold is compatible with distinct dynamic strategies, ranging from global restraint to controlled peripheral flexibility.

To further distinguish large-scale unfolding from temperature-dependent redistribution within the folded conformational ensemble, we projected the trajectories onto a two-dimensional space defined by backbone RMSD and the fraction of native contacts, Q (Fig 5). The resulting free-energy landscapes revealed distinct temperature-dependent conformational responses among the three enzymes.

**Fig 5:**
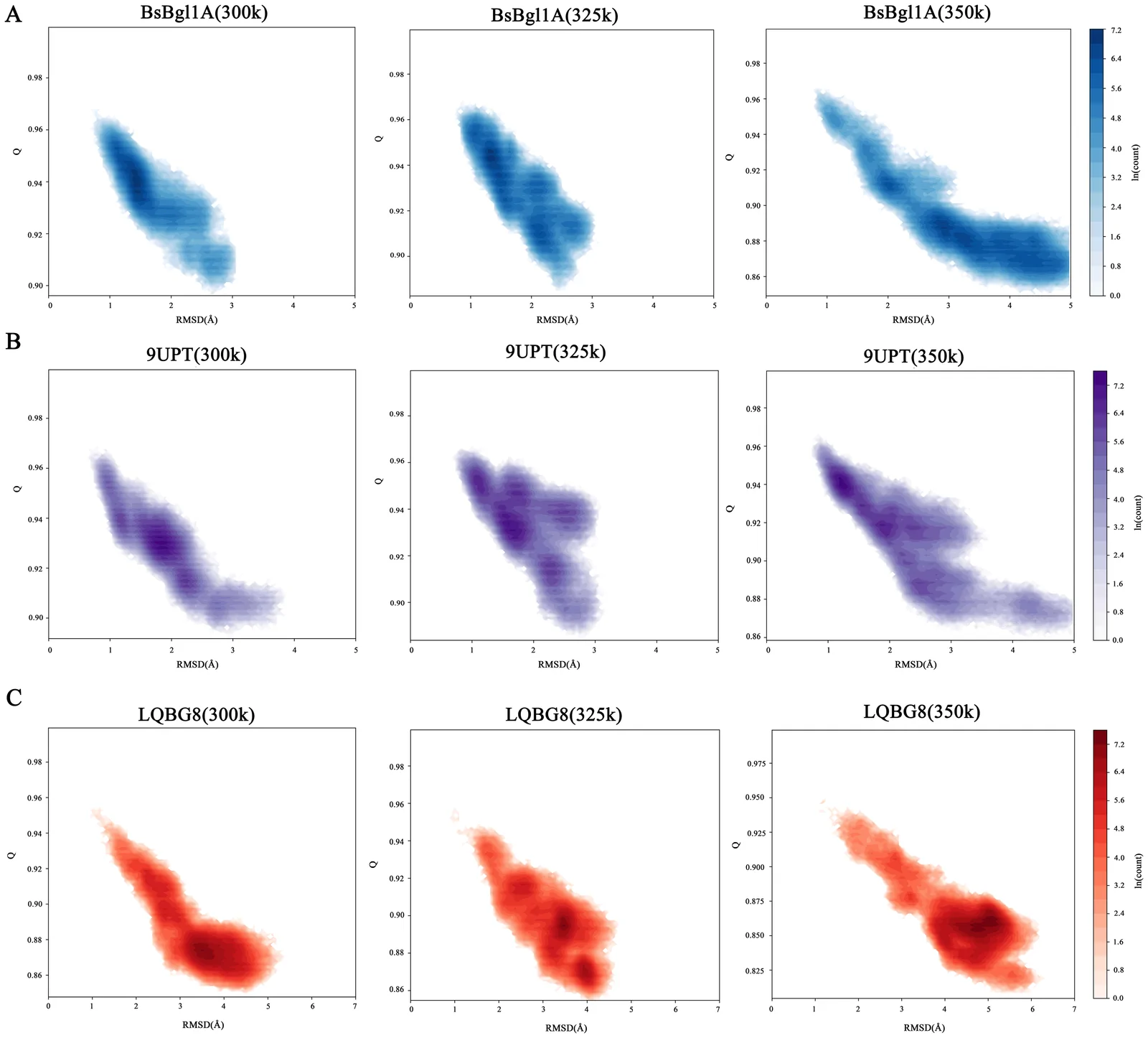
Temperature-dependent conformational landscapes of BsBgl1A, 9UPT, and LQBG8 projected onto backbone RMSD and native-contact fraction. Two-dimensional free-energy landscapes are shown for BsBgl1A **(A)**, 9UPT **(B)**, and LQBG8 (C) at 300, 325, and 350 **K.** The horizontal axis represents backbone RMSD relative to the corresponding initial structure, and the vertical axis represents the fraction of native contacts, **Q.** The color scale indicates the dimensionless free-energy-like quantity, − ln(P), calculated from the normalized conformational probability distribution; darker regions correspond to more highly populated conformational states. BsBgl1A exhibited a pronounced expansion toward a high-RMSD, low-Q basin at 350 K, whereas 9UPT retained a comparatively restrained conformational distribution across temperatures. LQBG8 sampled the broadest conformational space and shifted toward higher-RMSD and lower-Q states with increasing temperature, while retaining a substantial fraction of native contacts. The landscapes therefore indicate distinct balances between conformational mobility and native-contact preservation rather than uniform temperature-induced unfolding.

BsBgl1A sampled a relatively restricted conformational region at 300 K, with the dominant population distributed mainly over RMSD values of approximately 1–2.5 Å and Q values of approximately 0.91–0.96. At 325 K, the conformational ensemble became broader and extended toward lower-Q states, although the principal basins remained within a largely native-like region. A more pronounced redistribution occurred at 350 K, where BsBgl1A populated an extended high-RMSD, low-Q basin reaching RMSD values of approximately 4–5 Å and Q values of approximately 0.86–0.90. This shift indicates substantial structural reorganization and partial loss of native contacts at the highest temperature, consistent with the increased RMSD and residue flexibility observed in the conventional trajectory analyses. In contrast, 9UPT exhibited the most restrained temperature-dependent changes. At 300 K, its conformational ensemble was concentrated mainly within RMSD values of approximately 1–3 Å and Q values of approximately 0.90–0.96. Although additional substates emerged at 325 K, the major population remained within a comparatively compact region of conformational space. At 350 K, the landscape broadened toward higher RMSD and lower Q, but the dominant states remained more continuously distributed and showed less pronounced population of highly displaced conformations than BsBgl1A. These observations are consistent with the comparatively restrained backbone dynamics and temperature-insensitive structural behavior of 9UPT. LQBG8 displayed the broadest conformational landscape at all three temperatures. Even at 300 K, it sampled RMSD values extending to approximately 5 Å, with dominant conformations located at lower Q values than those of BsBgl1A and 9UPT. The landscape remained broadly distributed at 325 K and shifted further toward higher-RMSD, lower-Q conformations at 350 K, where the principal basin was centered near RMSD values of approximately 4–5.5 Å and Q values of approximately 0.84–0.88. Thus, LQBG8 does not achieve thermal adaptation through confinement within a rigid, low-RMSD conformational basin. Instead, it retains a broad and plastic conformational ensemble while preserving a substantial fraction of native contacts.

Taken together, the RMSD–Q landscapes indicate that the three GH1 enzymes remain within partially native-like conformational ensembles over the simulated temperature range, although the degree of structural redistribution differs markedly. BsBgl1A shows the strongest temperature-induced transition toward a distinct high-RMSD, low-Q basin at 350 K, whereas 9UPT maintains the most restrained conformational landscape. LQBG8 combines extensive conformational plasticity with persistent native-contact retention. These findings support the interpretation that thermal adaptation in these enzymes is not determined by uniformly low RMSD or maximal structural rigidity, but may involve distinct balances between conformational mobility, native-contact preservation, and higher-order dynamic organization.

To determine how the residue-contact architecture responds to increasing temperature, we calculated contact survival probabilities for all residue pairs across the three independent simulations of each enzyme. The resulting matrices revealed a highly conserved diagonal contact pattern in BsBgl1A, 9UPT, and LQBG8, reflecting the persistence of short-range interactions between sequentially adjacent residues and the overall preservation of the GH1 TIM-barrel framework (Fig 6). In addition to the main diagonal, recurrent off-diagonal contact clusters were observed between nonadjacent structural modules, indicating that the barrel architecture is maintained by a set of reproducible long-range residue contacts.

**Fig 6:**
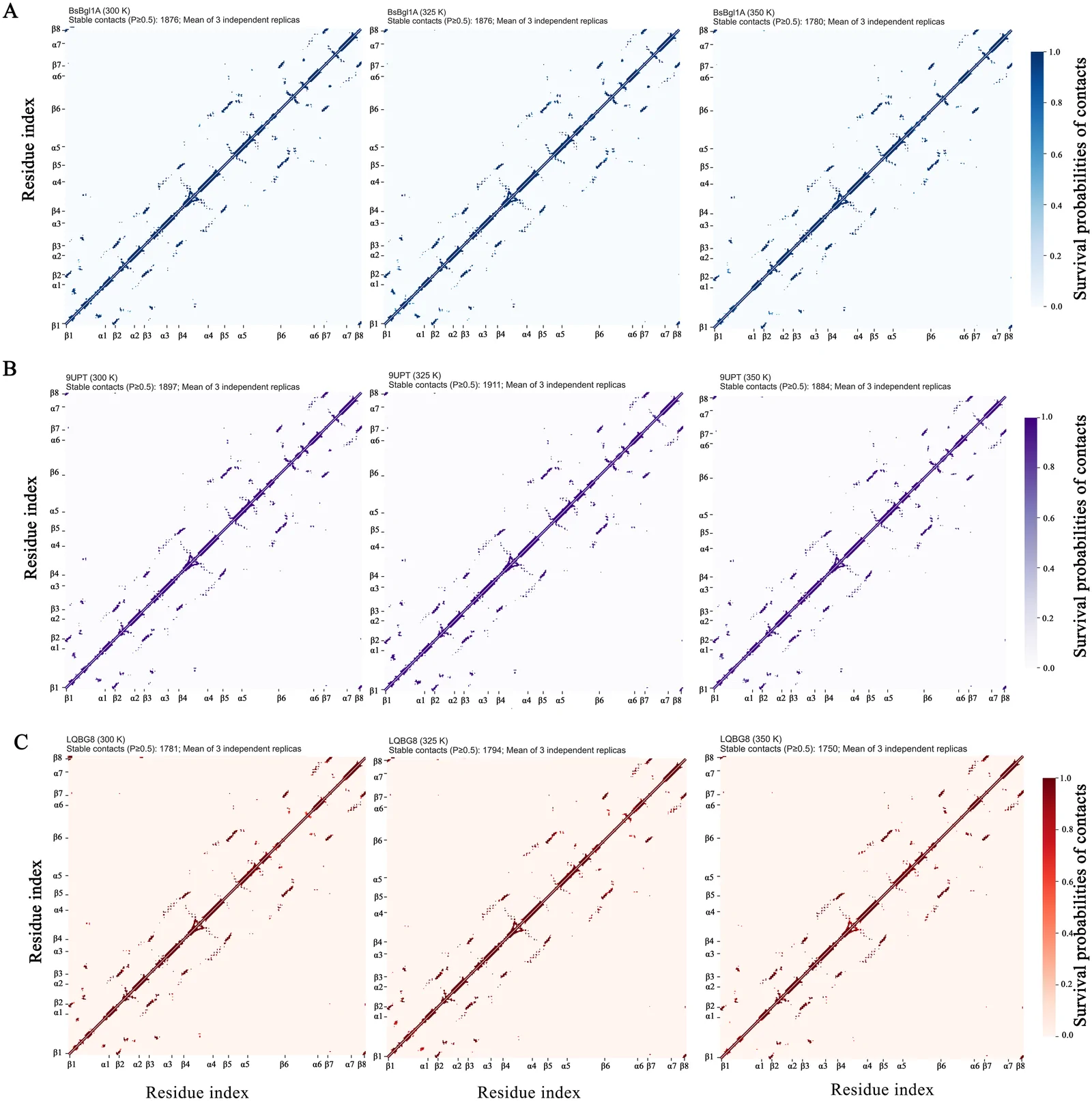
Residue contact survival probability maps of BsBgl1A, 9UPT, and LQBG8 at different temperatures. Contact survival probability matrices are shown for **(A)** BsBgl1A, (B) 9UPT, and **(C)** LQBG8 at 300, 325, and 350 K, arranged from left to right. Each matrix represents the mean contact survival probability calculated from three independent molecular dynamics simulations. Residues are ordered according to their positions in the protein sequence and annotated by the corresponding sequential βα structural modules of the GH1 TIM-barrel fold. Each matrix element represents the fraction of analyzed trajectory frames in which the corresponding residue pair satisfied the predefined native-contact criterion. Darker colors indicate higher contact survival probabilities, whereas lighter colors indicate less persistent or absent contacts. The main diagonal primarily reflects local contacts between residues that are close in sequence, whereas off-diagonal clusters represent contacts between sequence-distant residues or structural modules. The number of stable contacts with a mean survival probability of at least 0.5 is indicated above each matrix. Self-contacts were excluded from the stable-contact counts. Blue, purple, and red color scales denote BsBgl1A, 9UPT, and LQBG8, respectively.

BsBgl1A showed a broadly preserved contact pattern between 300 and 325 K, whereas a noticeable reduction in stable contacts was observed at 350 K. Although the principal diagonal and several major intermodule contact clusters remained detectable, many weaker peripheral contacts became less persistent at the highest temperature. This loss was distributed mainly outside the most strongly conserved contact blocks and is therefore more consistent with progressive weakening of peripheral and interfacial packing than with complete disruption of the TIM-barrel core.

In contrast, 9UPT exhibited the most temperature-invariant contact architecture. The number and spatial distribution of stable contacts remained similar across 300, 325, and 350 K, with the characteristic diagonal and off-diagonal interaction blocks preserved throughout the temperature series. A modest increase in stable contacts was observed at 325 K, followed by only a limited reduction at 350 K. These results are consistent with the restrained structural fluctuations observed in the conventional molecular dynamics analyses and suggest that 9UPT maintains a comparatively robust residue-contact framework under thermal perturbation.

LQBG8 also retained the principal contact topology across all three temperatures. Despite having fewer stable contacts than 9UPT, the major diagonal and long-range contact clusters remained spatially conserved from 300 to 350 K. The relatively small variation in the number of contacts exceeding the survival-probability threshold indicates that heating primarily redistributes the persistence of individual residue contacts rather than causing widespread collapse of the contact network. This behavior is compatible with the coexistence of substantial local flexibility and preservation of a globally integrated TIM-barrel scaffold.

Replicate-level quantification of the stable-contact population further supported these temperature-dependent differences (S5 Fig). BsBgl1A showed a progressive decrease in the mean number of stable contacts with increasing temperature, with the lowest values observed at 350 K. By contrast, 9UPT maintained a comparatively high contact count across all three temperatures, showing only a modest increase at 325 K and a limited decrease at 350 K. LQBG8 exhibited a non-monotonic response, with an increase in stable contacts at 325 K followed by a decline at 350 K. Although replicate-to-replicate variability increased under some elevated-temperature conditions, the overall trends were consistent with the corresponding contact survival maps. These results indicate that heating affects not only the number of persistent contacts but also their redistribution across the structural network.

Comparison among the three enzymes therefore indicates that thermal adaptation is not determined simply by the total number of stable residue contacts. Instead, the temperature dependence and spatial organization of those contacts appear to be more informative. BsBgl1A undergoes the largest high-temperature loss of stable contacts, whereas 9UPT maintains the most invariant contact map and LQBG8 preserves its major contact topology despite pronounced regional mobility. These findings support a model in which thermal resilience depends on selective retention of structurally important contact clusters within the TIM-barrel core, together with a greater tolerance for contact rearrangement in peripheral regions. Because contact survival describes persistence rather than interaction energy or causal stabilization, these results should be interpreted as evidence of differential structural organization rather than as a direct measurement of the energetic contribution of individual contacts.

### 3.3. Thermostability emerges from preservation of a distributed dynamic interaction network

The preceding analyses showed that BsBgl1A, 9UPT, and LQBG8 retained the overall GH1 TIM-barrel fold despite exhibiting distinct temperature-dependent conformational fluctuations. However, conventional structural metrics alone do not describe how statistically coordinated residue motions are organized across the protein scaffold. We therefore applied neural relational inference to the molecular dynamics trajectories to reconstruct directed residue–residue and module–module coupling networks for BsBgl1A, 9UPT, and LQBG8 at 300, 325, and 350 K. The three enzymes have reported optimal temperatures of approximately 45, 65, and 70 °C, respectively, providing a comparative series with distinct thermal phenotypes.

Before interpreting the spatial organization of the inferred networks, we examined the distributions of directed edge probabilities and their sensitivity to threshold selection (S7 and S8 Figs). The edge-probability distributions differed among the three enzymes and temperature conditions, indicating that the observed network differences were not generated solely by application of a common cutoff. At the selected threshold of 0.1, BsBgl1A showed a pronounced increase in the fraction of retained edges at 325 K, followed by a reduction at 350 K, whereas 9UPT and LQBG8 retained comparatively consistent populations of higher-probability edges across temperatures. Threshold-sensitivity analysis over the range of 0.02–0.30 reproduced the same qualitative temperature-dependent trends, although the absolute retained-edge fractions varied with the cutoff. Thus, the non-monotonic response of BsBgl1A and the comparatively persistent coupling patterns of 9UPT and LQBG8 were not restricted to a single arbitrarily selected threshold.

Quantitative network metrics further supported these distinctions (S9 Fig). BsBgl1A exhibited a marked increase in average total degree, weighted node strength, and directed network density at 325 K, followed by lower values at 350 K. In contrast, 9UPT showed comparatively modest temperature-dependent changes in these metrics, whereas LQBG8 retained relatively high network density and weighted node strength across the temperature series. These quantities describe the abundance and distribution of statistically inferred coupling and should not be interpreted as direct measures of physical stability, causal signal transmission, or energy transfer. In particular, the elevated connectivity of BsBgl1A at 325 K occurred together with increased conformational fluctuation and is therefore interpreted as temperature-dependent overcoupling or network reorganization rather than as enhanced structural stability.

Each protein was partitioned into eight sequential βα modules corresponding to the repeating structural units of the canonical TIM barrel (Fig 7A–7C, 7Ci). Although the exact residue boundaries varied slightly among the proteins, this segmentation enabled comparison of coupling patterns across equivalent architectural elements. At both the residue and module levels, the inferred interactions were not uniformly distributed throughout the structures (Fig 7A–7C, 7Cii–&7Ciii). Instead, prominent interaction bands and high-weight connections repeatedly involved the central βα5–βα7 region, identifying this sector as a recurrently coupled structural region across the three enzymes. To facilitate interpretation of these interaction patterns, the corresponding directed module-level networks are presented separately in Fig 8.

**Fig 7:**
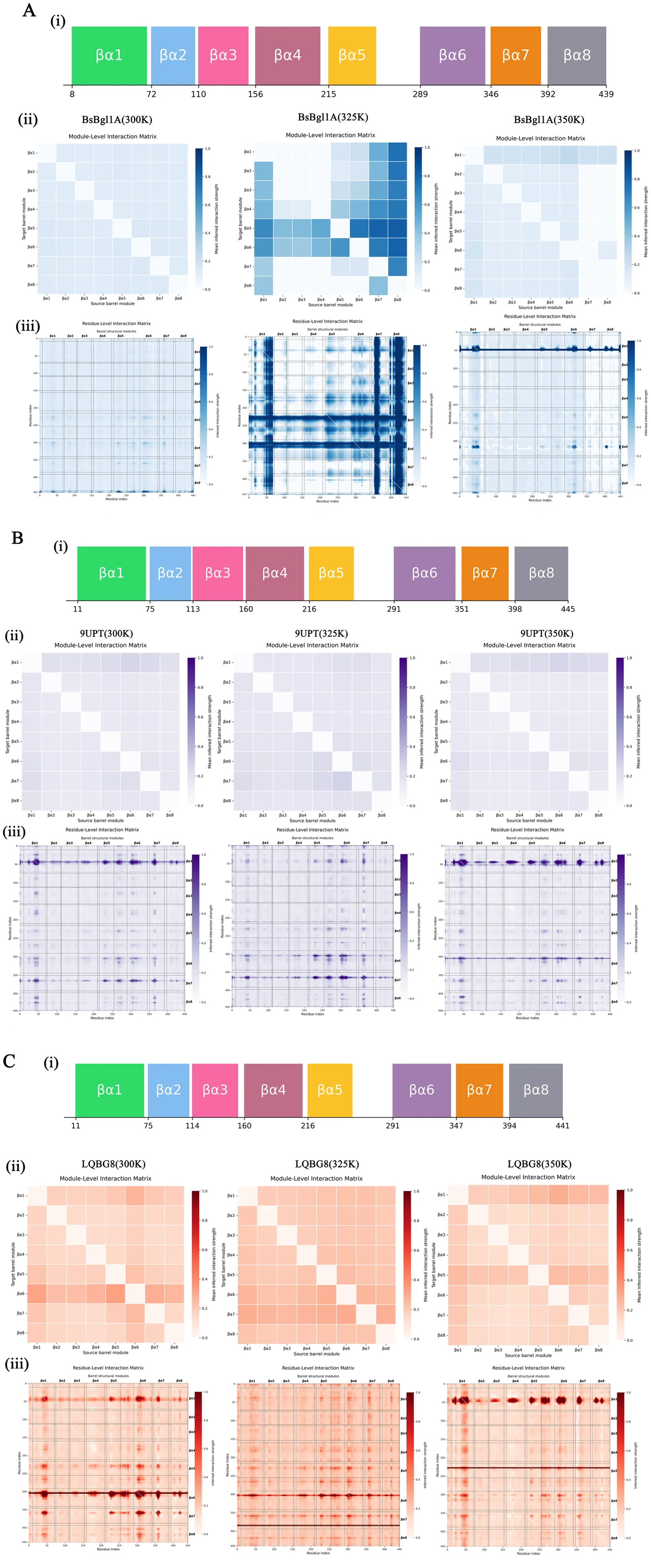
Temperature-dependent residue- and module-level dynamic interaction patterns of BsBgl1A, 9UPT, and LQBG8 inferred by neural relational inference. **(A–C)** Dynamic interaction analyses of BsBgl1A, 9UPT, and LQBG8, respectively, at 300, 325, and 350 **K. (i)** Segmentation of each protein into eight sequential βα structural modules corresponding to the repeating units of the GH1 TIM-barrel scaffold. Residue boundaries are indicated below the colored module diagrams. **(ii)** Module-level directed interaction matrices inferred from the molecular dynamics trajectories. Rows and columns represent target and source βα modules, respectively, and color intensity denotes the mean inferred interaction strength. **(iii)** Residue-level directed interaction matrices. Black lines delineate the boundaries of the eight βα modules, and darker colors indicate stronger inferred residue–residue coupling.

**Fig 8:**
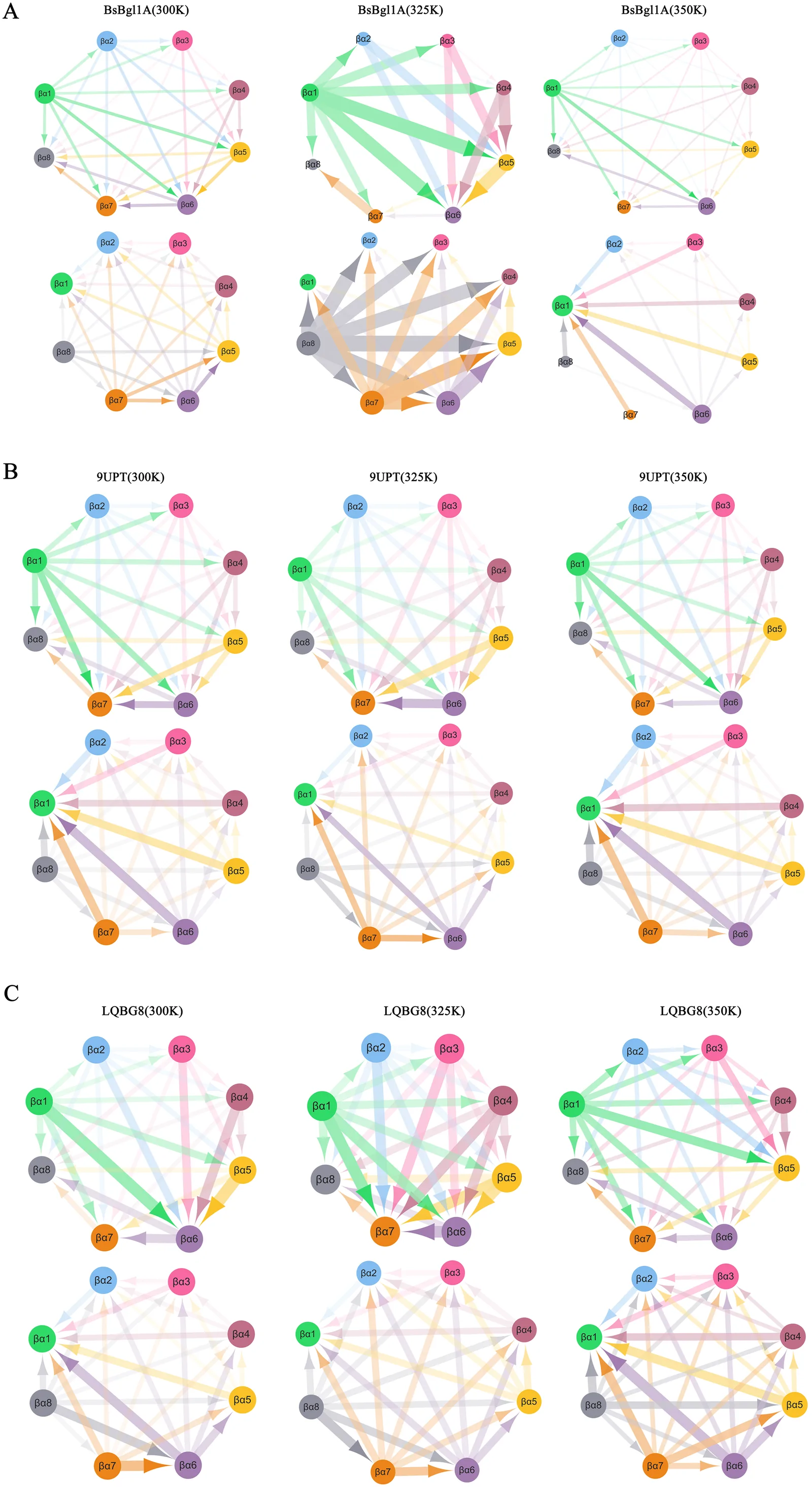
Temperature-dependent module-level dynamic interaction networks of BsBgl1A, 9UPT, and LQBG8 inferred by neural relational inference. **(A–C)** Circular representations of the directed module-level interaction networks of BsBgl1A, 9UPT, and LQBG8, respectively, at 300, 325, and 350 K. For each temperature, the upper and lower network diagrams visualize complementary outgoing- and incoming-oriented interaction patterns, respectively. Nodes represent the eight βα structural modules (βα1–βα8) and are arranged according to their sequential order in the TIM-barrel structure. Arrow direction denotes the inferred direction of communication, whereas edge thickness and opacity are proportional to the inferred interaction strength. Persistent connections involving the central βα5–βα7 modules identify this region as a conserved dynamic communication core. BsBgl1A exhibits transient network amplification at 325 K followed by a marked loss of connectivity at 350 K, whereas 9UPT and particularly LQBG8 retain more integrated network architectures under thermal stress.

At 300 K, BsBgl1A exhibited a relatively weak and diffuse module-level interaction matrix, with only modest coupling among the eight βα units (Fig 7A, ii). The residue-level matrix similarly showed sparse interaction bands, indicating that statistically coordinated motion was concentrated within a limited number of residue groups (Fig 7A, iii). Nevertheless, the corresponding directed network retained several coupling relationships involving the central βα5–βα7 modules (Fig 8A). At 325 K, BsBgl1A underwent a marked reorganization. Both the module- and residue-level matrices displayed a pronounced increase in inferred interaction strength, particularly for connections involving βα5, βα6, βα7, and βα8 (Fig 7A, ii–iii), while the directed network became dominated by several high-weight edges converging on or emerging from the C-terminal half of the barrel (Fig 8A). This increase should not be interpreted as evidence of improved stability. In the context of the elevated RMSD and RMSF observed for BsBgl1A, it more plausibly reflects a temperature-induced reorganization in which regions that were only weakly coordinated at 300 K became more strongly and broadly coupled at 325 K. This pattern was not maintained at 350 K. Instead, most module-level interactions weakened, residue-level coupling became sparser (Fig 7A, ii–iii), and the directed network showed reduced connectivity (Fig 8A). Although residual coupling involving βα1 and the βα5–βα7 sector remained, the network no longer exhibited the broadly distributed organization observed at 325 K. Thus, BsBgl1A showed a non-monotonic response characterized by weak basal coupling, pronounced intermediate-temperature overcoupling, and reduced connectivity at the highest temperature.

The inferred interaction organization of 9UPT was comparatively stable across the three temperatures (Figs 7B and 8B). Its module-level matrices showed relatively low absolute interaction weights at 300, 325, and 350 K, but the overall distribution of coupling changed only modestly (Fig 7B, ii). Residue-level interaction bands were retained at broadly similar sequence positions (Fig 7B, iii), indicating that comparable structural regions continued to participate in statistically coordinated motion. Consistent with these matrix-level patterns, the directed networks showed persistent coupling involving βα1 and the central-to-C-terminal modules, particularly βα5, βα6, and βα7 (Fig 8B). Unlike BsBgl1A, 9UPT did not show a large intermediate-temperature amplification followed by extensive network loss. Instead, it maintained a more restrained and comparatively temperature-insensitive coupling pattern. These observations suggest that thermal resistance in 9UPT is associated with preservation of a reproducible higher-order network organization rather than with uniformly high interaction strength. LQBG8 displayed the most consistently interconnected module-level organization across the simulated temperature range (Figs 7C and 8C). At 300 K, its interaction matrix already showed broadly distributed coupling among several βα modules, with comparatively strong interactions involving βα5 and βα6 (Fig 7C, ii). The residue-level matrix contained multiple continuous horizontal and vertical interaction bands (Fig 7C, iii), indicating statistically coupled motion among spatially separated regions. At 325 K, the interaction matrices showed that this broadly distributed organization was retained, with strengthened coupling involving the βα5–βα7 sector (Fig 7C, ii–iii). Correspondingly, the directed network contained multiple high-weight relationships linking these central modules with both N-terminal and C-terminal regions (Fig 8C). Importantly, this overall organization was largely retained at 350 K. Although individual edge weights and dominant module pairs were reorganized, the network did not undergo the widespread reduction in connectivity observed for BsBgl1A. The βα5–βα7 sector remained integrated within the directed network at all three temperatures. In particular, βα5 and βα6 repeatedly showed high incoming or outgoing coupling strength, whereas βα7 retained connections with more peripheral modules (Fig 8C). These observations indicate that LQBG8 accommodates substantial local conformational mobility while maintaining a broadly integrated pattern of statistically inferred long-range coupling.

To assess whether the inferred higher-order network organization was reproducible beyond the data used for model optimization, we separately reconstructed networks from the training and held-out test datasets using the trained encoder with fixed parameters (S10 Fig and S3 Table). The relative distribution of module-level coupling was generally preserved between the two datasets, although the absolute interaction strengths were consistently lower in the held-out networks. Module-level Spearman correlations ranged from 0.548 to 0.952 across the nine enzyme–temperature conditions, indicating moderate-to-strong agreement in the relative ranking of the eight βα modules. Agreement was condition dependent and was weaker for BsBgl1A at 325 K, consistent with the greater sampling sensitivity of its strongly reorganized network. Edge-level reproducibility was more variable than module-level agreement, indicating that individual inferred residue–residue edges were more sensitive to sampling variation than higher-order module organization. These results support interpretation of the NRI outputs primarily at the level of module-level patterns, residue importance, and temperature-dependent network trends, rather than as invariant physical pathways between specific residue pairs.

Taken together, the matrix-based interaction patterns in Fig 7 and their directed network representations in Fig 8 refine, rather than replace, the interpretation obtained from RMSD, Rg, RMSF, secondary-structure, and catalytic-site analyses. Greater thermal adaptation was not associated with uniform rigidification or uniformly stronger coupling. Instead, the three enzymes exhibited distinct organizations of correlated motion under thermal perturbation. Within this limited comparative set, the more thermally adapted enzymes were associated with greater persistence or reorganization of an integrated βα5–βα7-centered coupling pattern. However, these observations remain correlational and do not establish that the inferred network itself causally determines thermostability or constitutes an independent target of natural selection.

### 3.4. Evolutionary constraints anchor the dynamic network core under thermal stress

The preceding analyses showed that thermal robustness in GH1 β-glucosidases is associated with preservation of an integrated dynamic interaction network centered on the TIM-barrel core. In particular, the βα5–βα7 modules repeatedly emerged as major communication hubs in the neural relational inference networks of BsBgl1A, 9UPT, and LQBG8. We next asked whether this core-centered architecture arises solely from the physical organization of the TIM-barrel fold or whether it has also been maintained by long-term evolutionary selection.

To establish a structurally consistent family-level dataset, we initially collected 179 experimentally resolved GH1 β-glucosidase structures and evaluated their similarity to a selected representative reference structure (S2 Table). Structures were retained only when they satisfied both an RMSD cutoff of 3 Å and a TM-score cutoff of 0.8. Of the 179 structures examined, 178 passed both criteria, corresponding to a retention rate of 99.4%, whereas 1BGG was excluded because of its relatively large RMSD and low TM-score (S11 Fig). This result demonstrates that the canonical GH1 fold is exceptionally well conserved across the structural dataset and provides a consistent framework for family-level evolutionary analysis.

Despite this strong fold conservation, the sequence-identity matrix revealed extensive amino acid divergence across the GH1 dataset (Fig 9A). Most non-diagonal sequence pairs exhibited only low-to-moderate identity, whereas highly similar sequences were restricted to several localized clusters. Thus, the GH1 family preserves a common three-dimensional architecture while accommodating substantial sequence variation.

**Fig 9:**
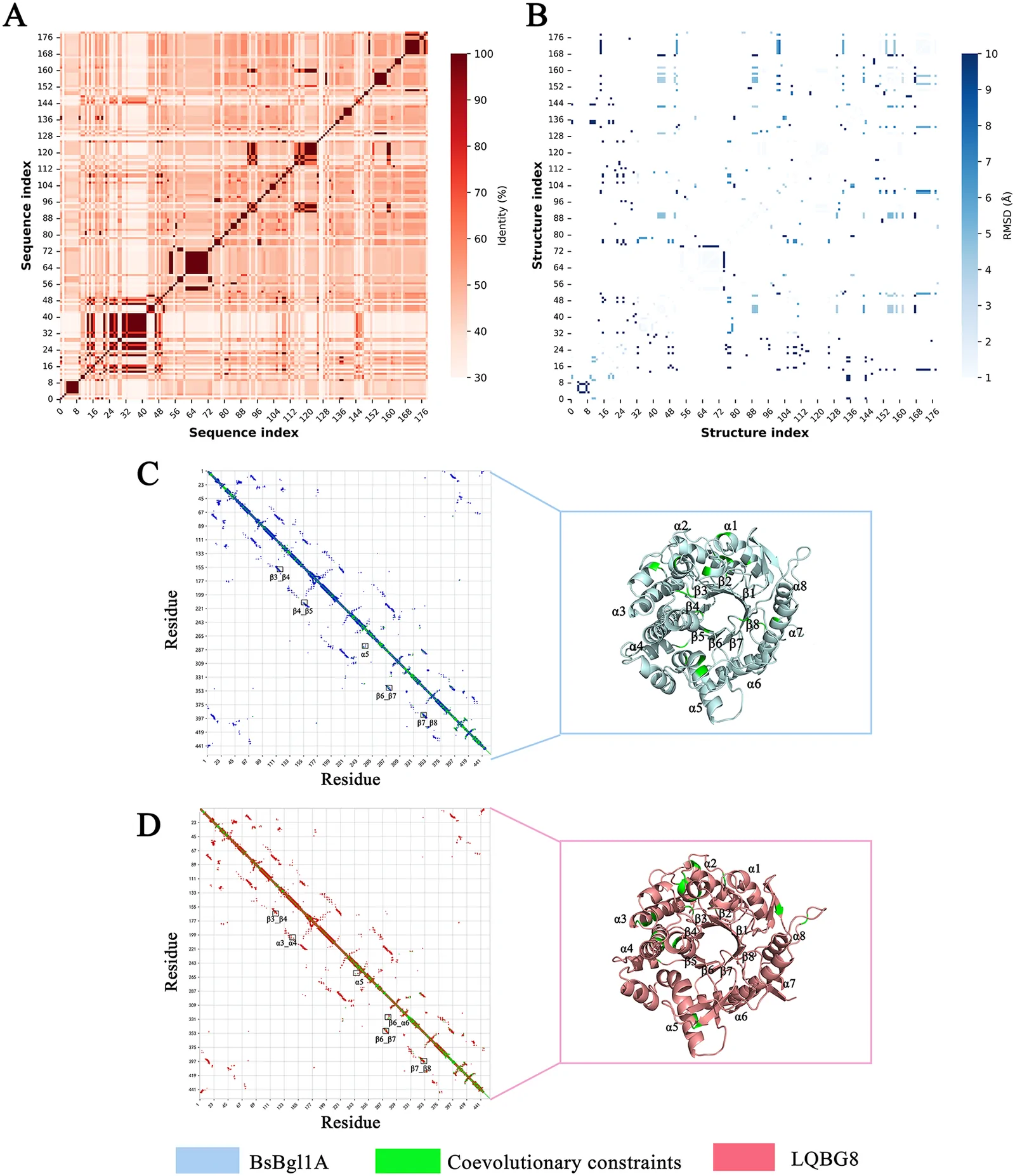
Family-level structural conservation and coevolutionary constraints in GH1 β-glucosidases. **(A)** Pairwise sequence-identity matrix for the structurally curated GH1 β-glucosidase dataset. Color intensity represents the percentage sequence identity between each pair of proteins. The heterogeneous off-diagonal pattern indicates extensive sequence divergence, with highly similar sequences restricted to localized clusters. **(B)** Pairwise structural root-mean-square deviation matrix for the corresponding GH1 structures. Color intensity represents backbone RMSD, with darker blue indicating larger structural deviation. The comparatively sparse distribution of high-RMSD pairs demonstrates that structural conservation is substantially greater than sequence conservation across the family. **(C, D)** Coevolutionary constraints mapped onto BsBgl1A and LQBG8, respectively. The left panels show residue-pair coupling maps, with βα module boundaries and representative secondary-structure elements indicated. Green signals denote inferred coevolutionary constraints. The right panels show the corresponding constraints mapped onto the three-dimensional structures. BsBgl1A and LQBG8 are shown in light blue and pink, respectively, and coevolutionarily constrained regions are shown in green. The enrichment of constraints around the central βα5–βα7 region identifies this sector as an evolutionarily conserved interaction core.

The corresponding structural comparison showed a much more conserved pattern than the sequence analysis (Fig 9B). Most structure pairs exhibited relatively small backbone deviations, although a limited number of regions displayed larger RMSD values. The contrast between the broadly divergent sequence matrix and the comparatively conserved structural matrix indicates that evolutionary constraints act strongly on maintenance of the TIM-barrel architecture rather than on uniform conservation of the primary sequence.

To determine how these constraints are distributed within the fold, we constructed a family-level multiple sequence alignment and inferred coevolving residue pairs. The resulting evolutionary couplings were then mapped onto the residue-contact frameworks and three-dimensional structures of BsBgl1A and LQBG8 (Fig 9C, 9D). The coevolutionary signals were highly non-uniform. Rather than being evenly distributed throughout the proteins, prominent coupling clusters occurred along and adjacent to the structural diagonal and were enriched around several barrel-associated regions, including the β3–β4, β4–β5, α5, β6–α6, β6–β7, and β7–β8 segments.

Importantly, many of these constrained regions converge spatially on the central βα5–βα7 sector. In both BsBgl1A and LQBG8, residues carrying strong coevolutionary constraints were concentrated around the inner barrel and the interfaces connecting βα5, βα6, and βα7. The similar spatial distribution observed in the two proteins indicates that this pattern is not specific to one sequence or one thermal phenotype, but reflects a conserved organizational feature of the GH1 family.

The localization of coevolutionary constraints closely coincides with the dynamic interaction architecture identified by neural relational inference. The βα5–βα7 modules that retained high connectivity and relay capacity under thermal perturbation also contain a substantial proportion of the family-level coevolving residue pairs. This correspondence suggests that the dynamic communication core is not simply an incidental consequence of residue packing within the TIM barrel. Instead, the residue couplings supporting this network appear to have been maintained during GH1 evolution.

By contrast, many peripheral βα loops and terminal regions displayed weaker or more dispersed coevolutionary signals. These regions also correspond to the portions of the structure that showed greater sequence-length variation, elevated RMSF, and stronger temperature-dependent network reorganization. The combined results therefore reveal a hierarchical evolutionary pattern: the peripheral regions retain sufficient plasticity to permit sequence and conformational diversification, whereas the central barrel network remains more strongly constrained.

The comparison between BsBgl1A and LQBG8 further suggests that conservation of the core network does not eliminate differences in thermal adaptation. Both proteins preserve the principal evolutionary couplings around βα5–βα7, but their ability to maintain the associated dynamic network at elevated temperature differs substantially. BsBgl1A undergoes transient overcoupling followed by network fragmentation, whereas LQBG8 preserves a dense, multi-route communication architecture. Thus, family-level evolution appears to conserve the location and basic organization of the dynamic core, while sequence-level adaptations modulate the resilience with which this core operates under thermal stress.

Taken together, these results establish a direct connection between structural conservation, residue coevolution, and temperature-dependent dynamic communication. The TIM-barrel core, particularly the βα5–βα7 sector, functions not only as a geometrically conserved scaffold but also as an evolutionarily constrained dynamic relay. Thermostability in GH1 β-glucosidases therefore appears to arise from preservation and refinement of a core-centered network that maintains long-range coordination while allowing peripheral regions to remain conformationally adaptable.

## 4. Discussion

Thermal adaptation requires proteins to maintain structural and functional integrity under intensified thermal fluctuations while retaining the conformational mobility necessary for substrate recognition and catalysis. Accordingly, thermostability is increasingly understood as a balance between stabilizing interactions and functionally required flexibility rather than as uniform rigidification of the entire molecular structure [43, 44]. By integrating comparative structural analysis, long-timescale molecular dynamics simulations, neural relational inference, and family-level coevolutionary analysis, the present study shows that GH1 β-glucosidases with a conserved TIM-barrel scaffold can exhibit distinct organizations of correlated motion under thermal perturbation. This association should not, however, be interpreted as an exclusive or direct causal mechanism of thermostability. Local packing, electrostatic interactions, regional rigidity modulation, solvent organization, protonation state, and the intrinsic mechanical properties of the TIM-barrel fold are likely to contribute jointly to the different thermal phenotypes of the three enzymes [45,46].

BsBgl1A, 9UPT, and LQBG8 all adopt the canonical GH1 (β/α)₈ TIM-barrel fold despite their different reported optimal temperatures of approximately 45, 65, and 70 °C, respectively. Their highly similar global architectures indicate strong structural constraint on the TIM-barrel scaffold. Structural divergence is instead concentrated predominantly in solvent-exposed loops and terminal regions of individual βα units, whereas the barrel-forming β-strands and their adjacent α-helices remain comparatively conserved. This pattern is consistent with the evolutionary plasticity of TIM-barrel proteins, in which a robust central scaffold can accommodate extensive sequence variation and structural remodeling in peripheral regions [47].

The three enzymes nevertheless displayed markedly different dynamic responses to temperature. BsBgl1A exhibited the most pronounced temperature-dependent increase in backbone deviation and residue-level mobility, particularly at 350 K. In contrast, 9UPT showed the lowest overall residue fluctuations at elevated temperature and the smallest fraction of residues exceeding an RMSF threshold of 2.0 Å, indicating a comparatively restrained conformational regime. LQBG8 displayed a distinct behavior: it exhibited relatively high RMSD values and extensive local flexibility even at lower temperatures, while retaining a compact global structure and preserving the principal secondary-structure elements of the TIM barrel. Thus, the greater thermal adaptation of LQBG8 cannot be attributed simply to lower RMSD or globally reduced flexibility.

The relatively stable radii of gyration of all three proteins provide important context for interpreting these differences. Although their RMSD and RMSF profiles differed substantially, none of the proteins underwent sustained large-scale expansion over the simulated temperature range. The RMSD–Q conformational landscapes further showed that the observed structural deviations primarily reflected redistribution within partially native-like conformational ensembles rather than complete unfolding (Fig 5). BsBgl1A sampled a relatively restricted conformational region at 300 K but shifted toward a distinct high-RMSD, low-Q population at 350 K, indicating pronounced structural reorganization and partial loss of native contacts at the highest temperature. In contrast, 9UPT maintained the most restrained conformational landscape, although its ensemble also broadened moderately with increasing temperature. LQBG8 sampled the broadest conformational space at all three temperatures and progressively shifted toward higher-RMSD and lower-Q states, while retaining a substantial fraction of native contacts and an overall compact structure. The elevated RMSD of LQBG8 is therefore more consistent with extensive sampling of alternative compact conformations than with sustained global expansion or complete unfolding. Together, these observations emphasize that conformational mobility and structural destabilization are not equivalent: large-amplitude fluctuations and partial native-contact rearrangement can occur without complete disruption of the folded scaffold [48].

Secondary-structure analysis further supports this interpretation. The central barrel architecture remained broadly preserved in all three enzymes, whereas temperature-dependent transitions among helical, strand, and loop conformations were concentrated mainly in peripheral and loop-rich regions. BsBgl1A exhibited the most pronounced increase in local structural heterogeneity at 350 K. 9UPT showed the greatest temporal continuity of its secondary-structure pattern, consistent with its comparatively restrained residue dynamics. LQBG8 also displayed local secondary-structure variability, particularly in regions corresponding to major RMSF peaks, but this variability was not accompanied by global disruption of the barrel scaffold. The thermal responses of these enzymes are therefore better described as differential redistribution of regional flexibility and native-contact organization than as a simple progression from rigidity to unfolding.

The catalytic-site analysis provides an additional functional context for these structural observations. The Cα–Cα distance between the two catalytic glutamate residues remained broadly stable across temperatures in all three enzymes, with only modest temperature-dependent shifts and substantial overlap among the three independent replicas (S6 Fig). Thus, the conformational redistribution observed in the RMSD–Q landscapes was not accompanied by pronounced disruption of the backbone-level spatial relationship between the catalytic residues. This result does not establish preservation of catalytic activity or side-chain reactive geometry, but it indicates that the major temperature-dependent differences in global and regional dynamics occurred while the overall backbone organization of the catalytic site remained largely intact.

The NRI analysis extends these observations by characterizing statistical coupling among residue motions. At both residue and βα-module resolutions, inferred interactions repeatedly involved the central βα5–βα7 region. However, the temperature dependence of this coupling differed substantially among the three enzymes. BsBgl1A exhibited relatively weak and diffuse coupling at 300 K, followed by a pronounced increase in inferred interaction strength at 325 K and reduced connectivity at 350 K. This non-monotonic behavior was supported by the edge-probability distributions and by changes in average node degree, weighted node strength, and directed network density.

The highly connected state of BsBgl1A at 325 K should not be interpreted automatically as enhanced stability. Increased statistical coupling can arise when previously semi-independent structural regions become more broadly synchronized under thermal perturbation. Such a state may represent compensatory reorganization or dynamic overcoupling rather than the formation of a more favorable or functionally efficient network. The reduction in connectivity at 350 K, together with the shift toward a high-RMSD, low-Q conformational population, is consistent with limited persistence of this intermediate-temperature coupling state. BsBgl1A therefore exhibits a temperature-sensitive and non-monotonic network response rather than simple progressive strengthening or weakening. 9UPT adopted a different dynamic strategy. Its inferred interaction strengths remained moderate, but their spatial distribution and module-level organization were comparatively reproducible across temperatures. No abrupt intermediate-temperature amplification followed by widespread network loss was observed. Together with its comparatively low RMSF and restrained RMSD–Q landscape at 350 K, these findings suggest that the thermal behavior of 9UPT is associated with maintenance of a relatively stable and restrained higher-order coupling pattern rather than with exceptionally strong interactions. LQBG8 exhibited the most consistently distributed network organization. Its residue- and module-level coupling patterns remained broadly connected across the temperature series, and the βα5–βα7 modules continued to participate in prominent coupling relationships. At 350 K, LQBG8 retained comparatively high directed network density despite substantial local residue mobility and a broad RMSD–Q landscape. This combination indicates that local flexibility and higher-order dynamic organization are not mutually exclusive. LQBG8 appears able to accommodate extensive regional motion and partial native-contact reorganization while retaining statistically coordinated relationships among spatially separated structural regions. Such an organization is compatible with the simultaneous requirements of thermal tolerance and conformational adaptability, although the present data do not demonstrate that the inferred coupling pattern directly preserves catalytic function.

The βα5–βα7 region represents the most consistent feature across the structural, dynamic, and evolutionary analyses. This region contains central elements of the TIM barrel, repeatedly participates in high-weight module-level coupling, and remains integrated within the high-temperature networks of 9UPT and LQBG8. Its prominence may partly arise from the intrinsic geometry, contact density, and mechanical centrality of the TIM-barrel scaffold. The current results therefore support two non-exclusive interpretations. First, βα5–βα7 may appear dynamically prominent because the central barrel is generically constrained by fold topology. Second, sequence-level adaptation may modulate the persistence or reorganization of correlated motion centered on this region. Distinguishing these contributions will require targeted perturbation and comparative control analyses.

Family-level analysis indicates that the same structural sector is also subject to long-term evolutionary constraint. After sequence-length and structural-similarity filtering, 178 GH1 structures were retained for coevolutionary analysis, confirming the high structural coherence of the analyzed family. Despite conservation of the fold architecture, the sequence-identity distribution revealed extensive sequence divergence. Coevolutionary couplings were non-uniformly distributed and enriched in barrel-associated regions, several of which converged spatially on the βα5–βα7 sector.

The correspondence between coevolutionary enrichment and NRI-derived dynamic prominence is consistent with the possibility that recurrent higher-order coupling patterns have been maintained during GH1 evolution. However, this correspondence does not constitute direct evidence that natural selection acts on the entire inferred network as a discrete selective unit. Coevolutionary couplings represent statistical dependencies among amino acid substitutions, whereas NRI-derived edges represent statistical dependencies among residue motions. Their overlap therefore remains correlational. Moreover, both signals may be independently enhanced in structurally central regions with high contact density. Residue-level permutation tests or structure-aware null models that preserve solvent accessibility, contact degree, sequence separation, secondary-structure class, and module membership will be required to determine whether the observed overlap significantly exceeds that expected from the geometry of the TIM-barrel core [49].

The conceptual contribution of this study is therefore not the identification of protein dynamic networks, long-range coupling, or allosteric organization, all of which are well established in protein science [50]. Rather, the results support a more focused hypothesis for GH1 β-glucosidases: enzymes sharing a conserved fold but possessing different thermal optima can organize correlated motions through distinct dynamic strategies, and greater thermal adaptation is associated with more persistent or reorganized integration of a βα5–βα7-centered coupling pattern under thermal stress. The intermediate behavior of 9UPT indicates that these dynamic patterns are not restricted to a binary comparison between BsBgl1A and LQBG8, while also revealing substantial mechanistic heterogeneity within the family.

Several limitations should be considered when interpreting these findings. First, NRI-derived probabilities describe statistical coupling learned from molecular dynamics trajectories rather than direct physical contacts, causal communication pathways, or energy-transfer mechanisms. Terms such as “dynamic interaction network,” “coupling pattern,” and “hub” are therefore used descriptively. Establishing mechanistic communication will require targeted mutagenesis, perturbation simulations, nonequilibrium analyses, or experimental measurements of energetic and functional consequences. Second, although the simulations comprised three independent 1 μs replicas at each temperature and the RMSD and Rg block analyses supported the reproducibility of the principal structural trends, convergence of global structural observables does not necessarily guarantee complete stationarity of the inferred networks. The held-out analysis showed that higher-order module organization was generally more reproducible than individual residue-level edges, but agreement remained condition dependent. Time-resolved NRI reconstruction, first-half versus second-half comparisons, block-specific network analysis, leave-one-replica-out validation, and repeated training with independent random initializations would provide a more stringent assessment of network stability, particularly for the highly connected BsBgl1A state at 325 K. Third, although the Cα–Cα distance between the two catalytic glutamate residues remained broadly stable across temperatures, this backbone-level metric does not directly demonstrate preservation of catalytic side-chain geometry, substrate-pocket organization, or enzymatic activity. Analyses of catalytic carboxyl-group distances and orientations, substrate-pocket solvent accessibility, pocket-volume distributions, ligand-bound trajectories, and experimental activity measurements will be required to establish a direct relationship between dynamic organization and functional resilience. This issue is particularly relevant to LQBG8, which combines pronounced conformational plasticity with persistent network connectivity. Fourth, although homology modeling based on experimentally resolved GH1 templates reduces dependence on uncertainties associated with de novo structure prediction, model uncertainty may remain in peripheral loops and local side-chain packing. Simulations initiated from alternative templates or independently generated structural models, together with local loop-quality comparisons, would provide an additional assessment of robustness. Finally, the present analysis is restricted to three GH1 β-glucosidases sharing a highly conserved TIM-barrel fold. The proposed interpretation should therefore be regarded as a testable hypothesis for GH1 enzymes and possibly related TIM-barrel proteins rather than as a universal model of thermophilic adaptation.

In conclusion, BsBgl1A, 9UPT, and LQBG8 organize conformational motion in distinct ways within a conserved TIM-barrel scaffold. BsBgl1A exhibits pronounced temperature sensitivity, a shift toward a high-RMSD, low-Q ensemble at 350 K, and a non-monotonic network response characterized by intermediate-temperature overcoupling followed by reduced connectivity. 9UPT maintains a comparatively restrained conformational landscape and persistent higher-order dynamic organization, whereas LQBG8 combines substantial conformational plasticity with sustained long-range statistical coupling and retention of a substantial fraction of native contacts. Across these different dynamic regimes, the βα5–βα7 region consistently emerges as a prominent structural, dynamic, and evolutionary sector. These findings support the hypothesis that thermal adaptation in GH1 β-glucosidases may involve not only local stabilization and modulation of rigidity, but also the persistence or reorganization of higher-order correlated dynamic patterns within the conserved barrel core.

## Supporting information

S1 Fig Structural comparison of AlphaFold2-derived and homology models of BsBgl1A and LQBG8. (TIF)

S2 Fig Temperature-dependent backbone stability of BsBgl1A, 9UPT, and LQBG8 revealed by molecular dynamics simulations. (TIF)

S3 Fig Temperature-dependent radius of gyration analysis of BsBgl1A, 9UPT, and LQBG8 during molecular dynamics simulations. (TIF)

S4 Fig Quantitative comparison of residue-level flexibility among BsBgl1A, 9UPT, and LQBG8. (TIF)

S5 Fig Replicate-level quantification of stable residue contacts in BsBgl1A, 9UPT, and LQBG8 at different temperatures. (TIF)

S6 Fig Temperature-dependent stability of catalytic-site geometry in the three GH1 β-glucosidases. (TIF)

S7 Fig Distribution and threshold sensitivity of inferred residue-level edge probabilities. (TIF)

S8 Fig Histograms of inferred directed-edge probabilities for the three GH1 β-glucosidases. (TIF)

S9 Fig Global graph properties of the inferred dynamic interaction networks. (TIF)

S10 Fig Module-level predictive performance of the Neural Relational Inference models. (TIF)

S11 Fig Structural similarity filtering of the GH1 β-glucosidase family. (TIF)

S1 Table Structural comparison of AlphaFold2-derived and homology-model starting structures. (XLSX)

S2 Table Experimentally resolved GH1 β-glucosidase structures included in the family-level analysis. (XLSX)

S3 Table Consistency of NRI-derived interaction networks between the training and held-out test datasets under frozen-model inference. (XLSX)
